# Preterm Birth and Antidepressant Medication Use during Pregnancy: A Systematic Review and Meta-Analysis

**DOI:** 10.1371/journal.pone.0092778

**Published:** 2014-03-26

**Authors:** Krista F. Huybrechts, Reesha Shah Sanghani, Jerry Avorn, Adam C. Urato

**Affiliations:** 1 Division of Pharmacoepidemiology and Pharmacoeconomics, Department of Medicine, Brigham and Women’s Hospital and Harvard Medical School, Boston, Massachusetts, United States of America; 2 Department of Obstetrics and Gynecology, Vanderbilt University, Nashville, Tennessee, United States of America; 3 MetroWest Medical Center, Tufts University School of Medicine, Framingham, Massachusetts, United States of America; Baylor College of Medicine, United States of America

## Abstract

**Introduction:**

Preterm birth is a major contributor to neonatal morbidity and mortality and its rate has been increasing over the past two decades. Antidepressant medication use during pregnancy has also been rising, with rates up to 7.5% in the US. The objective was to systematically review the literature to determine the strength of the available evidence relating to a possible association between antidepressant use during pregnancy and preterm birth.

**Methods:**

We conducted a computerized search in PUBMED, MEDLINE and PsycINFO through September 2012, supplemented with a manual search of reference lists, to identify original published research on preterm birth rates in women taking antidepressants during pregnancy. Data were independently extracted by two reviewers, and absolute and relative risks abstracted or calculated. Our *a priori* design was to group studies by level of confounding adjustment and by timing of antidepressant use during pregnancy; we used random-effects models to calculate summary measures of effect.

**Results:**

Forty-one studies met inclusion criteria. Pooled adjusted odds ratios (95% CI) were 1.53 (1.40–1.66) for antidepressant use at any time and 1.96 (1.62–2.38) for 3^rd^ trimester use. Controlling for a diagnosis of depression did not eliminate the effect. There was no increased risk [1.16 (0.92–1.45)] in studies that identified patients based on 1^st^ trimester exposure. Sensitivity analyses demonstrated unmeasured confounding would have to be strong to account for the observed association.

**Discussion:**

Published evidence is consistent with an increased risk of preterm birth in women taking antidepressants during the 2^nd^ and 3^rd^ trimesters, although the possibility of residual confounding cannot be completely ruled out.

## Introduction

Preterm birth is a major clinical problem throughout the world. It is the leading cause of infant mortality: approximately 75% of all perinatal deaths occur among preterm infants [1]. It is also a major contributor to both short- and long-term morbidity: surviving infants are at increased risk of health problems ranging from neurodevelopmental disabilities such as cerebral palsy and mental retardation to other chronic health problems such as asthma [2]. Although the risk is highest in very preterm infants (<32 gestational weeks), it has been well documented that moderate (32–33 gestational weeks) and mild (34 to 36 gestational weeks) preterm birth infants are also at increased risk for neonatal and post-neonatal mortality and morbidity [3]–[6]. Rates of preterm birth have been increasing over the past two decades and it is a major public health concern [7], with costs to society that have been estimated to be as high as $26.2 billion per year in the US [7], and £939 million per year in the UK [8]. It has been reported that two thirds of these costs are incurred for the care of babies born moderately prematurely [8].

In many developed countries, the use of antidepressant medications has increased sharply between 1996 and 2005, and now surpasses antihypertensives as the most commonly prescribed drug class in ambulatory care [9]. During this same time period, rates of antidepressant use during pregnancy have increased approximately 4-fold, with reported rates of up to 3–6% in Europe [10]–[12] and up to 8% in the US [13], [14].

Numerous studies, of varying size and quality, have examined the effects of antidepressant medication use on pregnancy outcomes, including preterm birth. They differ in terms of the timing of the antidepressant exposure during pregnancy and adjustment for potential confounding variables, including lifestyle factors, co-morbidities, and the severity of the underlying psychiatric illness. The extent to which such differences contribute to variability in findings remains to be elucidated. The objective of this review was to determine the strength of the available evidence relating to a possible association between antidepressant use during pregnancy and preterm birth, and to assess this relationship in terms of (1) the timing of the antidepressant use studied, and (2) attempts to control for the possible confounding effects of depression itself.

## Methods

To identify all available studies on the topic of antidepressant medication use during pregnancy and preterm delivery, we performed a computerized search in PUBMED, MEDLINE, and PsycINFO using the key words: (“antidepressant*” or “tricyclic antidepressant*” or “selective serotonin reuptake inhibitor*” or “serotonin-norepinephrine reuptake inhibitor*”) and (“preterm birth*” or “preterm deliver*” or “pregnanc*” or “pregnancy complication*”). The databases were searched from their inception through September 12, 2012. Reference lists of selected articles were also searched to identify additional studies that reported on preterm births and antidepressant exposure.

Studies were included if they identified a group of pregnant women exposed to antidepressants at some point during their pregnancy as well as a comparison group, and reported on preterm birth rates, irrespective of whether preterm birth was a pre-specified study endpoint or one of several pregnancy characteristics reported. No restrictions were imposed on study size or design.

Once relevant studies were identified, for each study two investigators with clinical and epidemiologic expertise (ACU, KFH) independently abstracted preterm birth rates in the group(s) exposed to antidepressants and in the comparator group(s). When these were not expressed as percentages in the manuscript, they were calculated. Relative risks (expressed as odds ratios in all studies) were also taken directly from the manuscript. Whenever available, preference was given to relative risks adjusted for potential confounding variables. When relative risks were not reported in the manuscript, we estimated the unadjusted odds ratio and its corresponding 95% confidence limits (Wald method) based on the available information. When relative risks were only presented graphically, we contacted the authors to obtain the corresponding numerical estimates. Types of antidepressants used, numbers of users and other pertinent exposure information (e.g., time and duration), and potential confounders accounted for were also retrieved. Any discrepancies in data abstraction were resolved by consensus between the reviewers.

A funnel plot was examined for evidence of publication bias [15]. Between-study heterogeneity was examined using the Cochran Q and I^2^ tests [16]. We used a random-effects meta-analysis model to calculate summary measures of effect while accounting for heterogeneity across studies [17]. Because of the critical importance of (1) the timing of antidepressant use during pregnancy, and (2) the potential for confounding by the presence of depression itself (confounding by indication), we had determined *a priori* to group studies by timing of antidepressant exposure and level of confounding adjustment. We chose this approach to address the inherent issues of clinical and methodological diversity [18]. Finally, we conducted a sensitivity analysis to identify the strength of the residual confounding that would be necessary to fully explain the estimated association between antidepressant medication use and preterm birth [19].

## Results

**Figure 1:**
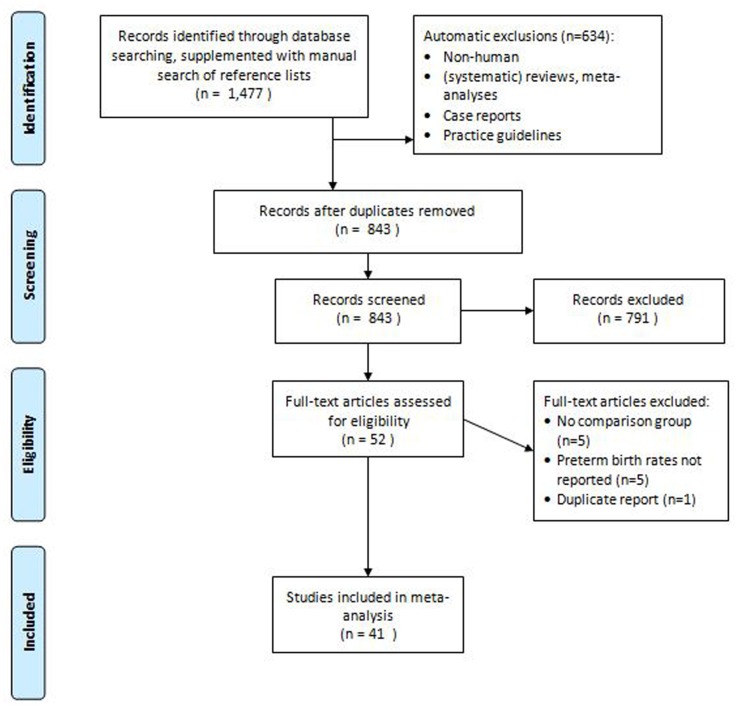
Study selection flowchart.

After an initial screen of the 1,477 studies identified through database searches, we identified 52 studies that met our predetermined criteria as possibly assessing the association between antidepressant use during pregnancy and preterm birth [20]–[67] [68]–[71]. Five studies reported on preterm births in antidepressant-exposed pregnancies but had no comparison group, and were excluded [20]–[24]. In five other studies a comparison was made between patients treated with antidepressants and untreated controls, but preterm birth rates were not reported [25]–[29]. One study used the same cohort [67] for which preterm birth rates had previously been reported [57]. (Figure 1).

Forty-one studies [30]–[66], [68]–[71], published between 1993 and 2012, were identified that met entry criteria as reporting on preterm birth rates in a group exposed to antidepressants versus a control group (Table 1). All were observational cohort studies; not surprisingly, no randomized controlled trials have been performed on this topic. Most studies (n = 21) were prospective in nature, four were bi-directional with some women included during pregnancy and some post-delivery, and the remaining 16 were retrospective (i.e., participants were identified after delivery). The majority of the retrospective studies (n = 14) used administrative healthcare utilization databases. Nine studies recruited patients through Teratogen Information Services, 17 recruited participants from clinics, physician offices and other referrals, and the remaining 15 studies used population-based electronic healthcare databases (with and without linkage to birth registries). The studies ranged in size from 44 to 1,618,255 participants. As expected, the studies using electronic healthcare databases were much larger (median: 199,547 participants) than those using other approaches (median: 290 participants).

**Table 1:** Characteristics of 41 studies evaluating the association between antidepressant medication use during pregnancy and preterm birth.

Author	Year	Study type	Exposure	Total N	Preterm birth rate					OR	95% CI
					Exposed		Reference				
Calderon-Margalit [30]	2009	Clinic-based prosp	Psychotropics,including SRI	2,793		12.1%		9.4%	Any time	1.21	(0.67; 2.21)
									T2/3	4.79	(1.66; 13.9)
Casper [31]	2003	Clinic-based prosp/retrosp	SRI	44		3.2%		7.7%	Any time	0.40	(0.02; 6.93)
Casper [32]	2011	Clinic-based prosp/retrosp	SRI	55		9.8%		0.0%		undefined	
Chambers [33]	1996	TIS prosp	fluoxetine	388		14.3%	T1/2	4.1%	T3 vs. T1/2	4.80	(1.10; 20.80)
							control	5.9%			
Colvin [34]	2011	Population-based healthdataset, linked tobirth registry	SRI	96,698		11.5%		8.0%	Any time	1.43	(1.24; 1.65)
Costei [35]	2002	TIS prosp	paroxetine	109		20.0%		37.0%	T3 vs. (T1/2or unexp)	6.50	(1.37; 30.91)
Davis [36]	2007	Population-based healthdataset	SRI, TCA	81,527	SRI	9.4%		6.6%	SRI - any time	1.45	(1.25; 1.68)
					TCA	1.1%			TCA - any time	1.67	(1.25; 2.22)
Diav-Citrin [37]	2008	TIS prosp	paroxetine,fluoxetine	1,953	paroxetine	8.7%		6.4%	paroxetine –T1&beyond	1.38	(0.91; 2.10)
					fluoxetine	9.0%			fluoxetine –T1&beyond	1.44	(0.90; 2.32)
Djulus [38]	2006	TIS prosp	mirtazapine,other ADs	312	mirtazapine	9.6%		1.9%	mirtazapine	5.43	(1.16; 25.41)
					other AD	6.7%			other AD	3.68	(0.75; 18.15)
Einarson [39]	2010	TIS prosp	all ADs	1,856		8.8%		5.4%		1.70	(1.18; 2.45)
Einarson [63]	2011	TIS prosp	all ADs	267	>1 AD	12.4%		4.5%	>1 AD	3.13	(0.95; 10.31)
					1 AD	10.1%			1 AD	2.39	(0.71; 8.07)
El Marroun [64]	2012	Population-based prosp	SRI	7,696	SRI	10.1%		5.1%	SRI	2.14	(1.08; 4.25)
					Depression,no tmt	6.3%			Depression,no tmt	1.10	(0.77; 1.59)
Ericson [40]	1999	National birth registry	all ADs	281,728		notprovided			T1&beyond	1.43	(1.14; 1.80)
Ferreira [41]	2007	Clinic-based retro	SRI,venlafaxine	166		27.6%		8.9%	T3	2.40	(0.9; 6.3)
Gavin [42]	2009	Prosp	ADs, otherpsychoactivemeds	3,019		notprovided			no depression	1.40	(0.8; 2.4)
									depression	1.40	(0.7; 2.7)
Grzeskowiak [68]	2012	Clinc-based retro	SRI	33,791		24.9%	psych illness	11.8%	SRI late vs.psych illness	2.68	(1.83; 3.93)
							no psych illness	10.7%	SRI late vs.no psych illness	2.46	(1.75; 3.50)
Hayes [70]	2012	Population-based healthdataset	All ADs	228,876	≥3 Rx filled	14.6%	no depression	13.5%	≥3 Rx vs.no Rx, 1^st^ trim	1.11	(0.94; 1.32)
							depression	13.5%	≥3 Rx vs. noRx, 2^nd^ trim	2.33	(1.96; 2.86)
									≥3 Rx vs. noRx, 3^rd^ trim	0.20	(0.15; 0.26)
Kallen [43]	2004	National birth registry	all ADs	563,656		10.3%		5.1%	all ADs - late	1.96	(1.6; 2.41)
									SRI - late	2.06	(1.58; 2.69)
									TCA - late	2.50	(1.87; 3.34)
Kieler [62]	2012	Population-based healthdataset	SRI	1,618,255		5.4%		3.8%		1.44	(1.37; 1.51)
Klieger-Grossman [61]	2011	TIS prosp	Escitalopram,other SRI	637	escitalopram	8.9%		4.2%	escitalopram –T1&beyond	2.21	(0.98; 5.00)
					other SRI	4.7%			other SRI –T1&beyond	1.12	(0.44; 2.81)
Latendresse [44]	2011	Clinic-based prosp	SRI	100		30.8%		5.7%		11.70	(2.2; 60.7)
Lennestal [45]	2007	National birth registry	SRI, SNRI/NRI	860,215	SNRI/NRI	9.1%		4.4%	SNRI/NRI –early	1.60	(1.19; 2.15)
					SRI	6.7%			SSRI - early	1.24	(1.11; 1.39)
Lewis [46]	2010	Clinic-based prosp	SRI, SNRI,NaSSA	54		14.8%		3.7%	continuous exposure	4.52	(0.47; 43.41)
Lund [47]	2009	Clinic-based prosp,linked to birth registry	SRI	57,001		8.8%	psych illness	5.0%	SRI any time vs.psych illness	2.05	(1.28; 3.31)
							no psych illness	4.9%	SRI any time vs.no psych illness	2.02	(1.29; 3.16)
Maschi [48]	2008	Clinic-based prosp	SRI, TCA	1,400	any time	6.5%		2.9%	any time	2.31	(1.14; 4.63)
					throughoutpregnancy	10.3%		2.6%	throughoutpregnancy	4.35	(1.31; 14.07)
Mulder [49]	2011	Clinic-based prosp	SRI	263		8.3%	psych illness	5.4%	SRI vs.psych illness	1.59	(0.32; 7.87)
							no psych illness	0.0%	SRI vs.no psych illness	undefined	
Nordeng [65]	2012	Population-based,linked to birth registry	all ADs,SRI	63,395	Any AD	6.6%		4.5%	Any AD	1.21	(0.87; 1.69)
					SRI	6.5%			SRI	1.28	(0.9; 1.84)
									Depression	1.13	(1.03; 1.25)
Oberlander [50]	2006	Population-based healthdataset	SRI	199,547		9.0%	depression	6.5%	SRI vs.depression: unadjusted	1.42	(1.17; 1.72)
									SRI vs.depression: adjusted	1.11	(0.75; 1.64)
							no depression	5.9%	SRI vs.no depression	1.59	(1.33; 1.91)
Pastuszak [51]	1993	TIS prosp	fluoxetine	170		7.1%		8.2%		0.85	(0.27; 2.63)
Pearson [52]	2007	Clinic-based retro	SRI, TCA	252		10.7%		10.1%		1.07	(0.45; 2.50)
Reis [53]	2010	National birth registry	all ADs	1,062,190	SRI	7.4%		5.5%		1.46	(1.31; 1.63)
					SNRI	10.0%				1.98	(1.49; 2.63)
					TCA	11.1%				2.36	(1.89; 2.94)
Roca [66]	2011	Clinic-based prosp/retro	SRI	252		13.1%		4.8%	exp vs. unexp	3.44	(1.3; 9.11)
									high vs. low dose	5.53	(1.46; 20.93)
Rurak [54]	2011	Clinic-based prosp	SRI	74		13.8%		2.2%		7.04	(0.75; 66.50)
Simon [55]	2002	Population-basedhealth dataset	SRI, TCA	788	SRI	10.3%		3.2%		4.38	(1.57; 12.22)
					TCA	10.0%		5.3%		1.86	(0.83; 4.17)
Sivojelezova [56]	2005	TIS prosp	SRI	396	any time;50% T1–T3	8.0%		3.8%		2.20	(0.81; 5.96)
Suri [57]	2007	Prosp (clinic and otherreferrals)	All ADs	90	>50% pregnancy	14.3%	depression	0.0%	depression history	notdefined	
							no depression	5.3%	no depression	3.00	(0.34; 26.19)
Toh [58]	2009	Retro	SRI,non-SRI	5,961	non-SRI	15.3%		7.3%	non-SRI	2.23	(1.02; 4.88)
					SRI - all	8.9%			SRI - all	1.12	(0.64; 1.95)
					SRI - continuersbeyond T1	10.5%			SRI - continuersbeyond T1	1.27	(0.59; 2.76)
					SRI –discontinuers	7.5%			SRI - discontinuers	1.01	(0.47; 2.19)
Wen [59]	2006	Population-based healthdataset	SRI	4,850		19.3%		12.0%		1.57	(1.28; 1.92)
Wisner [60]	2009	Prosp (clinic and otherreferrals)	SRI	238		15.5%	depression	13.9%	SRI vs. depression	1.14	(0.36; 3.56)
							no depression	6.1%	SRI vs. no depression	2.82	(1.08; 7.37)
Wogelius [71]	2006	Population-based healthdataset	SRI	151,831	SRI - early	7.2%		5.0%		1.48	(1.17; 1.87)
					SRI –early & late	8.8%				1.83	(1.33; 2.54)
Yonkers [69]	2012	Prosp (clinic and otherreferrals)	SRI	2,654	SRI –depression	16.4%	depression	10.2%	SR depr vs. nodepression	1.51	(0.60; 3.80)
					SRI –no depression	11.3%	no depression	7.8%	SRI no depr vs. nodepression	1.50	(0.94; 2.40)
									Depression vs. nodepression	0.86	(0.44; 1.70)

Abbreviations: Prosp = Prospective cohort; Retro = Retrospective cohort; depr = depression; AD = antidepressant; SRI = serotonin reuptake inhibitor; TCA = tricyclic antidepressant; NaSSA = Noradrenergic and specific serotonergic antidepressants; SNRI = Serotonin–norepinephrine reuptake inhibitors; NRI = noradrenaline reuptake inhibitors; T = trimester; exp = exposed; TIS = Teratogen Information Service.

All but one study defined preterm birth as an infant born before 37 weeks’ gestation, in accord with the WHO definition. Maschi et al considered infants born before 36 weeks of gestation as premature [48]. Most studies evaluated the association between selective serotonin-reuptake inhibitors (SSRI) and preterm birth (n = 22), but some also evaluated other antidepressants such as tricyclic antidepressants (TCA) and serotonin–norepinephrine reuptake inhibitors (SNRI) (n = 17). Two studies evaluated the effect of a variety of psychotropic medications, including antidepressants.

Table 2 presents the findings from the heterogeneity tests, along with the summary effect estimates from the meta-analysis. Medium to high heterogeneity was found across studies that adjusted for potential confounding factors (I^2^∶46 to 85%), but not across studies that provided unadjusted estimates (I^2^<25%). We conducted a random-effects meta-analysis of the adjusted estimates given the consistency in the direction of the effects [18].

**Table 2:** Effect of antidepressant medication use during pregnancy on preterm birth: meta-analysis results.

Level of adjustment	Timing ofexposure	Number of individualstudy estimates	Summary OR(95% CI)	Heterogeneity		
				Q_df_	(P value)	I^2^(95% uncertaintyinterval)(4)
Unadjusted	Early(1)	8	1.57 (1.30–1.90)	8.09_7_	(0.324)	13.5 (0.0–56.2)
	Any time	4	1.44 (1.34–1.56)	2.87_3_	(0.411)	0.0 (0.0–84.0)
Adjusted for potential confounders(3)	Early	8	1.16 (0.92–1.45)	46.47_7_	(<0.001)	84.9 (72.1–91.9)
	Late(2)	12	1.96 (1.62–2.38)	69.26_11_	(<0.001)	84.1 (73.8–90.4)
	Any time	17	1.53 (1.40–1.66)	19.72_16_	(0.233)	18.9 (0.0–54.3)
Adjusted for psychiatric illness						
Controls with psychiatric illness	All combined	12	1.61 (1.26–2.05)	20.47_11_	(0.039)	46.3 (0.0–72.5)
Controls without psychiatric illness	All combined	7	1.88 (1.48–2.40)	7.46_6_	(0.280)	19.6 (0.0–63.2)

(1) Typically 1st trimester; some women continued during pregnancy, others discontinued.

(2) Typically 3^rd^ trimester.

(3) Factors varied between studies, but typically included maternal age, smoking, alcohol use, parity, and history of prematurity or miscarriage.

(4) Values of I^2^ are percentages (% of variance explained). 95% uncertainty intervals are calculated as proposed by Higgins and Thompson [89].

### Unadjusted Estimates

**Figure 2:**
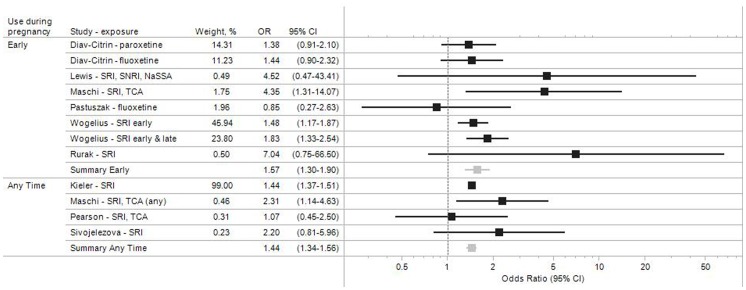
Study-specific and pooled odds ratio estimates for antidepressant medication during pregnancy and preterm birth. Studies that did not adjust for other risk factors.

Figure 2 summarizes the results for the 9 studies that did not account for potential confounding factors, either by design or because preterm birth rates were not pre-specified study endpoints. The category ‘*Early*’ includes studies in which women were known to have taken antidepressants early in pregnancy, typically in the first trimester. Some of these women continued antidepressant medication use during pregnancy whereas others discontinued. Studies classified under ‘*Any time*’ are those in which women were considered exposed irrespective of the specific time during pregnancy that medication was used, and studies where the timing was not specified. If multiple exposures were analyzed in a given study (e.g., estimate for paroxetine and fluoxetine [37]), they have all been included to ensure completeness of the evidence presented.

In these unadjusted analyses, the pooled odds ratio for the risk of preterm birth following antidepressant use in pregnancy was 1.57 (95% CI 1.30–1.90) for early exposure, and 1.44 (1.34–1.56) for exposure any time during pregnancy (Figure 2, Table 2). With the exception of two studies [51], [52], the unadjusted point estimates for all other studies suggest that antidepressant medication use during pregnancy may be associated with an increased risk of preterm delivery, but the effects are estimated imprecisely in many studies, as evidenced by the width of the 95% confidence intervals.

### Estimates Adjusted for Potential Confounders

**Figure 3:**
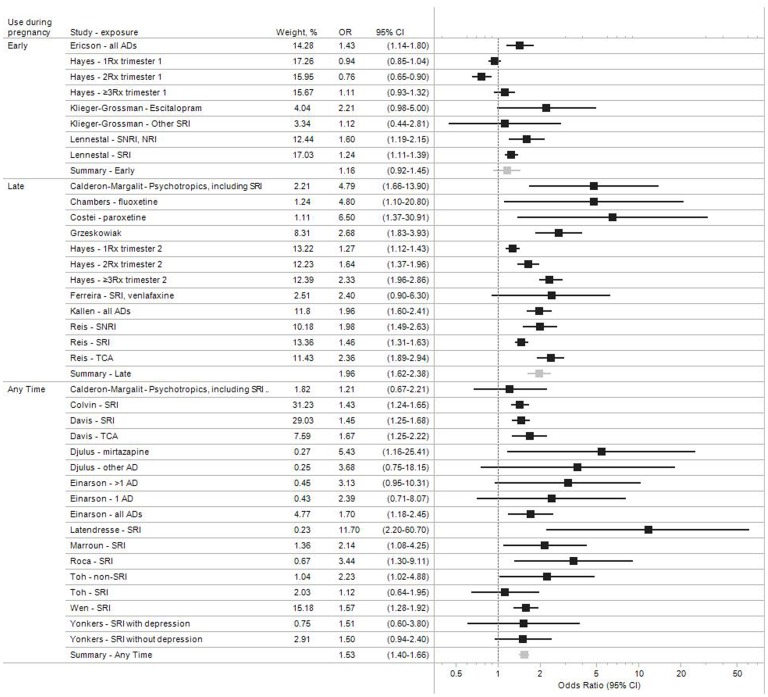
Study-specific and pooled odds ratio estimates for antidepressant medication during pregnancy and preterm birth. Studies that adjusted for other risk factors.

The adjusted odds ratios for the 22 studies which accounted for potential confounding variables are shown in Figure 3. The potential confounding factors adjusted for varied between studies, but typically included maternal age, smoking, alcohol use, parity, and history of prematurity or miscarriage.

In addition to the previously defined ‘*Early*’ and ‘*Any Time*” categories defining the timing of antidepressant use, Figure 3 also includes a ‘*Late*’ category which comprises studies in which women were known to use antidepressant medications late in pregnancy, generally in the third trimester. Results suggest an increased risk of preterm birth for all exposure-outcome combinations, with four exceptions. Calderon-Margalit and colleagues [30] found an increased risk for use of SSRIs late in pregnancy (aOR, 95%CI = 4.79, 1.66–13.90), but a much weaker association for exposure at any time (1.21, 0.67–2.21). Toh et al [58] estimated a positive association for non-SSRI antidepressants (2.23, 1.02–4.88), but not for SSRIs (1.12, 0.64–1.95). Klieger-Grossman et al [61] found a positive association for escitalopram (2.21, 0.98–5.00), but not for all other antidepressants combined (1.12, 0.44–2.81). Hayes et al [70] observed a positive association and a dose-response relation with second trimester exposure, but not with first trimester exposure.

In general, associations appeared stronger for antidepressant use known to have occurred late in pregnancy (pooled aOR, 95% CI: 1.16, 0.92–1.45 for early exposure; 1.53, 1.40–1.67 for exposure at any time; and 1.96, 1.62–2.38 for late exposure) (Figure 3, Table 2).

### Estimates Adjusted for Psychiatric Illness

**Figure 4:**
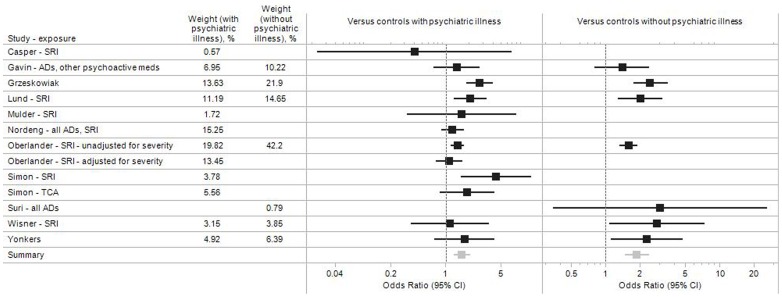
Adjusted study-specific and pooled odds ratio estimates for antidepressant medication during pregnancy and preterm birth. Subset of studies that account for the underlying psychiatric illness.

A major concern about the validity of studies assessing the effect of antidepressant medications on preterm birth is the potential for confounding by indication. It has been hypothesized that the underlying depression and its severity, or the behaviors potentially associated with depression (e.g., smoking, alcohol intake, nutritional changes), rather than antidepressant medication, may themselves increase the risk of preterm birth [72]. A few studies tried to address this concern directly by using as a comparator group women with a diagnosis of depression or other psychiatric illnesses who did not use antidepressant medications during their pregnancy, or by adjusting for the presence of a psychiatric diagnosis (Figure 4). Most of these 11 studies nonetheless found an increased risk of preterm birth associated with antidepressant medication use, resulting in a pooled OR of 1.61 (95% CI 1.26–2.05) for antidepressant users compared to women with psychiatric illness but no antidepressant use, versus 1.88 (1.48–2.40) compared to women without psychiatric illness or antidepressant use (Figure 4, Table 2). Oberlander et al [50] found an increased risk of preterm birth for women taking antidepressant medication compared to untreated women with a depression diagnosis (1.42, 1.17–1.72), but the association was much attenuated in a subgroup matched on depression severity (1.12, 0.75–1.64). Wisner and colleagues [60] found an increased risk for women on SSRI treatment (OR = 2.82), which was similar in magnitude to the increased risk seen in women with a depression diagnosis who were untreated (OR = 2.48), both compared to untreated women without depression. There were substantial differences, however, between SSRI users and non-users in terms of socio-economic status, alcohol use, and history of preterm birth, with SSRI users having consistently worse histories in these domains. These differences likely contributed to the equally high preterm birth rate observed among women with untreated depression (>20%), and were not accounted for in the analyses which only adjusted for age and race.

A few studies indirectly addressed the issue of confounding by depression by comparing preterm birth rates in women who continued vs. those who discontinued their antidepressant medication during pregnancy. Comparing women who continued antidepressant use through the third trimester with those who discontinued, Chambers and colleagues estimated an OR of 4.8 for fluoxetine [33]. Toh et al observed an increased risk of preterm birth in women who continued SSRI treatment beyond the first trimester (OR = 1.27), but not in those who discontinued their medication before the end of the first trimester (OR = 1.01), although the CI were wide and largely overlapped (Table 1) [58].

### Sensitivity Analyses

**Figure 5:**
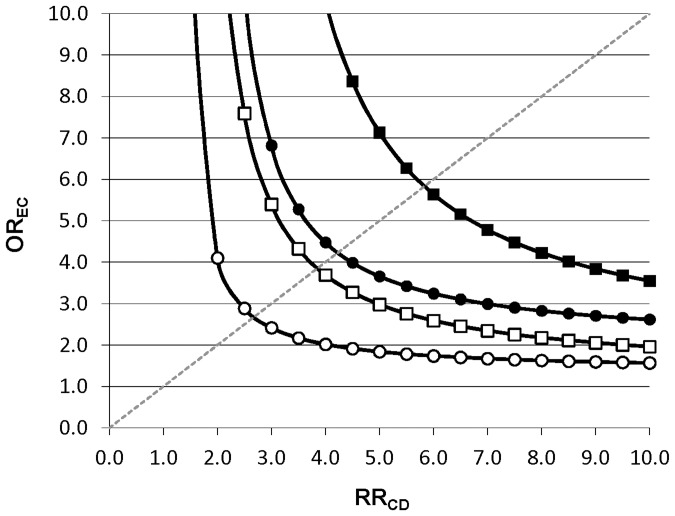
Sensitivity analysis of residual confounding (Rule-out approach). Example for estimated OR = 1.61 (depression adjusted point estimate) and OR = 1.26 (lower 95% bound of depression adjusted estimate) for different levels of confounder prevalence (▪ P_c_ = 0.05, OR = 1.61; •P_c_ = 0.25, OR = 1.61; □ P_c_ = 0.05, OR = 1.26; ○P_c_ = 0.25, OR = 1.26). Each line splits the area into two. The upper right area represents all combinations of OR_EC_ and RR_CD_ that would create confounding by an unmeasured factor strong enough to move the point estimate of OR to the null (OR = 1) or beyond. The area to the lower left represents all parameter combinations that would not be able to move the estimated OR to the null.

Figure 5 displays the strength of the association between a potential unmeasured confounder and the exposure (OR_EC_) and the outcome (RR_CD_) that would be required to fully explain the observed increased rate of preterm birth associated with antidepressant medication use during pregnancy (depression-adjusted OR = 1.61) if in truth no such increase existed. For an unmeasured confounder present in 25% of the population, relative risks ≥4 linking the hypothetical confounder to both antidepressant medication use and preterm birth would need to be present to fully explain the observed association, assuming 8% of pregnant women are exposed to antidepressants [14]. For a confounder present in just 5% of the population, relative risks >5.5 would be needed. For an apparent association of 1.26 (lower bound of the 95% CI for the depression-adjusted OR), the required strength would be >2.5 for an unmeasured confounder present in at most 25% of the population.

**Figure 6:**
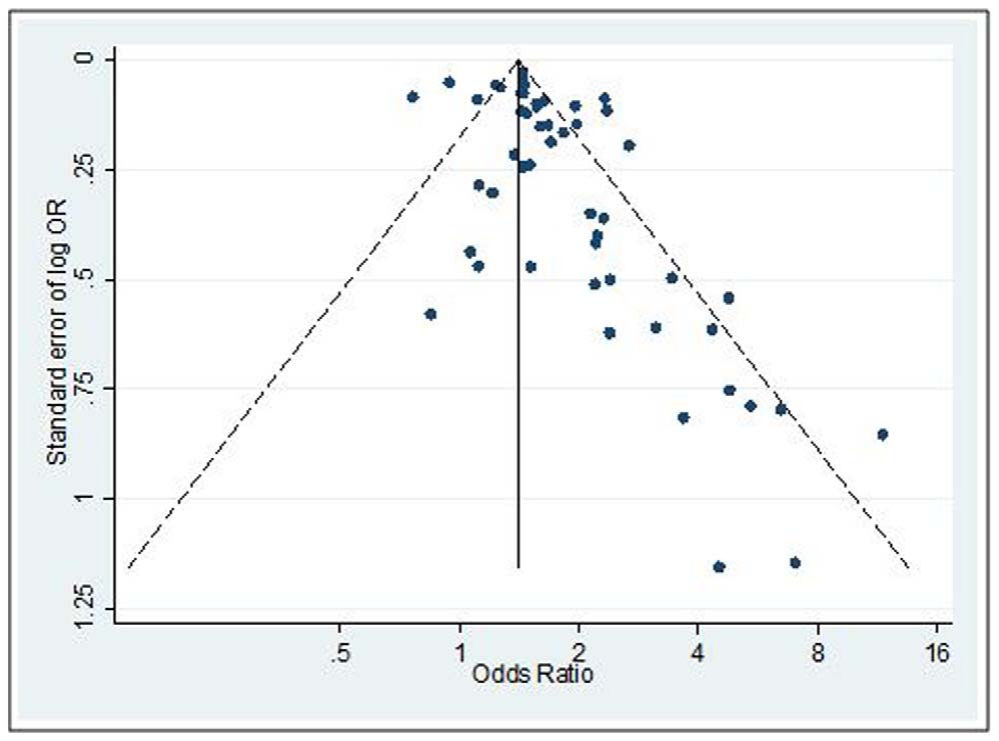
Funnel plot with pseudo 95% confidence limits.

Visual inspection of the funnel plot reveals some asymmetry, suggesting smaller studies with negative associations might be under-represented in the literature (Figure 6). It should be noted, however, that funnel plot asymmetry need not result from bias [15].

## Discussion

This systematic review of the literature concerning the relationship between antidepressant use and preterm birth identified 41 observational studies. Findings suggest the risk of preterm birth is increased in women treated with antidepressant medications during pregnancy, with pooled odds ratio estimates ranging between 1.16 (95% CI 0.92–1.45) and 1.96 (95% CI 1.62–2.38). The associations were stronger for antidepressant use later in pregnancy. Adjusting for a diagnosis of depression in most cases did not eliminate the effect, although the strength of the observed associations was somewhat attenuated. Sensitivity analyses demonstrated that very strong risk factors of preterm birth that are fairly imbalanced among exposure groups and independent of the adjusted confounders must be unmeasured and uncontrolled to explain the observed associations. Although it would be unlikely to miss such a strong single confounder, it is conceivable that several weaker confounders could have acted together to account for the apparent effect. Our findings are consistent with those from an earlier study which evaluated the association between prenatal antidepressant exposure and a range of adverse pregnancy and delivery outcome [73].

Preterm birth is a major problem worldwide [74]. The rate of antidepressant use during pregnancy has steadily increased over time in many industrialized nations, from less than 1% of women exposed in the early 90s to 3–6% in 2006 in Europe [10], [12], and to 7.5% in 2008 in the US [13]. It is therefore essential to determine if antidepressant use increases the risk of preterm birth. Studies in this area, however, are complicated due to several issues.

First, antidepressant exposure in many pregnancies is not a “yes or no” phenomenon. Many women stop antidepressants when they discover they are pregnant, resulting in first trimester exposure only. Others stop, but may restart the medications later in the pregnancy. Still other patients do not take the medications for much of the pregnancy but start in the third trimester. The issue of classifying exposure is therefore complex. Several studies, for example, have been performed by Teratogen Information Services (TIS). Women who called the TIS and reported that they were taking antidepressants were classified as “exposed” in those studies. The advantages of this design include the fact that exposure is determined prior to outcome, eliminating recall bias, and a control group is readily identifiable–women who contact the TIS with exposure to another medication. Yet, there are also major drawbacks to this approach. Most women who contact TIS are concerned about the use of antidepressants during pregnancy; therefore many of them may not continue with the medication throughout the pregnancy. If exposure to antidepressants in the second and third trimesters is more likely to be associated with preterm birth (and available evidence suggests that this might be the case) then TIS studies that classify women as exposed solely on the basis of first trimester exposure would likely underestimate the association between antidepressant use and preterm delivery. Studies that rely on electronic healthcare databases, on the other hand, contain detailed information on filled prescriptions for antidepressant medications during the entire pregnancy. Automated pharmacy dispensing information is usually seen as the gold standard of drug exposure compared to self-reported information [75] or prescribing records in outpatient medical records [76]. Patient recall bias is not an issue in healthcare utilization databases since all data recording is independent of a patient’s memory or agreement to participate in a research study [77]–[80]. However, filling a prescription does not necessarily guarantee that it was ingested, which could result in some misclassification. Such misclassification of exposure which is independent of the outcome status is likely to bias results towards the null (i.e. attenuate the association between antidepressant use and preterm birth.).

A major concern is the potential for confounding by the underlying depression and its severity, and associated poor health behaviors (e.g., nutrition, smoking, drug and alcohol use). More severely depressed women may be more likely to take antidepressants during pregnancy, and it has been suggested that it may be the depression itself that is causing the preterm birth and not the medication. Several of the studies in this systematic review made efforts to control for maternal depression and these studies continued to show increased rates of preterm birth in the antidepressant exposed pregnancies. The majority of studies we reviewed did not find increased preterm birth rates in depressed women unexposed to medication. However, despite these studies’ attempts to control for depression, it is likely that women with a diagnosis of depression who opt to continue treatment during pregnancy are inherently different from women with a depression diagnosis who discontinue treatment during pregnancy. It is therefore questionable whether these studies completely addressed confounding by indication severity. Nevertheless, the available data on whether depression itself is associated with preterm birth is inconsistent [81], and expert review panels have concluded that there is no clear association between depression and preterm birth. The Institute of Medicine reviewed this question and concluded that “Overall, recent prospective studies on depression do not suggest a strong pattern for depression as a general risk for preterm delivery, consistent with the results of earlier studies” [7]. A 2009 AAP/ACOG review similarly concluded: “Available data neither support nor refute a link between MDD [major depressive disorder] and these outcomes [preterm delivery and gestational age]” [82]. Despite the weak evidence to support the independent association between depression and adverse pregnancy outcomes, such as preterm birth, the prior belief is strong among some investigators [83].

Some limitations of this review, resulting from limitations in the source data, should be noted. The studies included were heterogeneous in terms of design, size, exposure assessment, timing and nature of the exposure, and confounding adjustment. For example, in some studies, women classified as exposed were only those taking medication throughout the pregnancy, and controls were those who stopped antidepressant use before the second trimester, while in others the “exposed” were patients taking an antidepressant in the first trimester, who may have stopped by the second trimester. We tried to address this by presenting the results separately by level of adjustment and timing of the exposure, but assignment of studies to these categories is somewhat subjective, and heterogeneity within categories remains. Although we would have liked to simultaneously investigate the effects of some of these factors through meta-regression, this was not feasible due to an insufficient number of studies. Given that all but one study defined preterm birth as a dichotomous outcome (<37 gestational weeks), we were not able to examine the strength of the association between antidepressant medication use and very-, moderate- and mild-preterm birth respectively. Nevertheless, even late preterm birth is a significant contributor to poor neonatal outcomes [3]–[6]. Likewise, the available evidence did not permit evaluation of the association with specific types of prematurity [83].

The most rigorous method for determining an association between antidepressant medication and preterm birth would be a randomized controlled trial, and some have argued for this [84]. Yet, numerous studies are now available that suggest antidepressant use during pregnancy may be associated with spontaneous abortion [85], birth defects [86], persistent pulmonary hypertension of the newborn [87], and newborn behavioral syndrome [88]. Whether it would be ethical to randomize depressed women to a medication arm with these possible effects is open for debate. Since it is unlikely a randomized trial will ever be conducted and since causality can never be ‘proven’ in the absence of trial data, we need to decide at what point the evidence is sufficiently strong to warrant informing patients, providers, and the public about the potential risks, so that they can be weighed against any expected benefits in a given patient.

In conclusion, the findings from our review of the literature are consistent with an association between antidepressant use during pregnancy and preterm birth, although the possibility of residual confounding by depression severity cannot be completely ruled out based on the available evidence. Counseling of pregnant women must take into consideration the clinical circumstances of a given patient, the strength of the available evidence on the risks and benefits (i.e., avoidance of risks associated with untreated depression), and alternatives to medication use during pregnancy. While our study findings cannot prove causality, they reinforce the notion that antidepressants should not be used by pregnant women in the absence of a clear need that cannot be met through alternative approaches.

## Supporting Information

Checklist S1 **Preferred Reporting Items for Systematic Reviews and Meta-Analyses (PRISMA) checklist.**
(DOC)
